# Supplementary material for: Non-Linear Modeling of Motor Development in Typically Developing Children and Youth Aged 5–18 Years Using Robot-Based Behavioral Assessments
Source: Bioengineering (Basel). 2025 Nov 12;12(11):1240. doi: 10.3390/bioengineering12111240 (PMC12649721; doi:10.3390/bioengineering12111240)
Supplement: Supplementary file 1 [file bioengineering-12-01240-s001.zip › bioengineering-3929190-supplementary.pdf]

Supplementary Material

This document contains supplementary material for the article, “Non-linear modelling of motor development in typically developing children and youth aged 5-18 years using robotic assessments”. The document contains tables of the results of the curve fitting for all parameters that were fit from the Visually Guided Reaching and Object Hit Kinarm Standard Tasks.

Tables S1 and S2 show the curve fitting results for the Visually Guided Reaching and Object Hit tasks, respectively. The six parameters highlighted in Figure 4 of the manuscript have been bolded in the tables. The fit type (exponential or quadratic) is represented by an “E" or “Q" superscript beside the parameter name in the table. All parameters were normally distributed without the need for transformation to achieve a Gaussian distribution of the z-scores. The r-squared values for the fitted curve ranged from 0 to 0.831 and 0 to 0.239 for the fit to the absolute values of the residuals for these six parameters.

R-squared values ranged from 0 to 0.835 for curve fits, and 0 to 0.357 for the residuals.

Table S1: Curve fitting results for the Visually Guided Reaching task.

Parameter Names	Curve Fitting to Data						Curve Fitting to Residuals			Measures of Normality	
	a_0	a_1	a_2	a_3	a_4	R- squared	m	b	R-squared	Skew	Kurtosis
Posture Speed^{E}*	0.133	-0.001	0.000	0.000	-0.127	0.130	0.000	0.003	0.113	0.748	3.218
**Reaction Time^{E}**	**1.152**	**-0.265**	**-0.003**	**-0.009**	**0.260**	**0.774**	**-0.003**	**0.068**	**0.239**	**0.552**	**2.945**
Initial Direction Angle^{E}*	0.106	-0.153	0.008	-0.003	0.045	0.248	-0.001	0.031	0.142	0.683	3.124
Initial Distance Ratio^{E}	-0.284	-0.100	-0.004	0.002	0.941	0.235	-0.003	0.094	0.070	0.133	3.054
Initial Speed Ratio^{E}*	-0.164	-0.244	0.000	-0.001	0.989	0.227	-0.002	0.041	0.155	-1.034	3.113
Speed Maxima Count^{E}	11.721	-0.641	-0.021	0.010	2.179	0.072	-0.004	0.334	0.016	0.327	3.077
Movement Time^{E}	1.364	-0.284	-0.062	-0.038	0.900	0.268	-0.001	0.121	0.006	0.391	3.190
**Path Length Ratio^{E}**	**0.935**	**-0.216**	**0.046**	**-0.038**	**1.162**	**0.447**	**-0.007**	**0.175**	**0.176**	**0.502**	**3.094**
Max Speed^{E}	-0.103	-0.178	0.021	0.001	0.176	0.185	0.001	0.024	0.037	0.448	2.868
**Min-Max Speed Difference^{E}**	**0.020**	**-0.045**	**0.003**	**0.00**	**0.004**	**0.168**	**0.000**	**0.007**	**0.013**	**0.587**	**3.040**
Note: superscripts E and Q mean an exponential or quadratic curve was fit to the date, respectively. Note that * indicates no transform created a Gaussian distribution of the residuals so the initial fit was used for simplicity.											

Table S2: Curve fitting results for the Object Hit task.

Parameter Names	Curve Fitting to Data						Curve Fitting to Residuals			Measures of Normality	
	a_0	a_1	a_2	a_3	a_4	R- squared	m	b	R-squared	Skew	Kurtosis
**Target Hits Total[E]**	**-386.146**	**-0.136**	**7.726**	**--**	**260.598**	**0.831**	**0.072**	**15.801**	**0.007**	**-0.265**	**2.894**
Median Error[E]	-37.217	-0.132	0.160	--	72.959	0.695	0.111	1.527	0.143	0.021	2.594
Miss Bias[Q]	0.000	0.002	0.003	--	-0.008	0.067	0.001	0.009	0.039	0.048	2.720
**Hand Bias of Hits[Q]**	**0.000**	**-0.007**	**0.002**	**--**	**0.130**	**0.000**	**-0.001**	**0.080**	**0.002**	**0.063**	**2.812**
Targets Hit (Dominant Hand)[E]	-195.657	-0.121	5.301	--	145.237	0.777	0.239	8.251	0.051	-0.220	2.871
Targets Hit (Non-Dominant Hand)[E]	-173.384	-0.142	3.864	--	115.481	0.732	0.248	7.635	0.016	0.047	2.534
Hand Speed (Dominant Hand)[E]	0.195	-0.010	0.019	--	0.028	0.121	-0.002	0.070	0.120	0.443	3.298
Hand Speed (Non-Dominant Hand)[E]	2.336	0.000	0.013	--	-2.142	0.073	-0.002	0.063	0.123	0.387	3.079
Hand Speed Bias[Q]	0.001	-0.017	0.008	--	0.151	0.069	0.000	0.068	0.014	-0.089	2.888
Movement Area (Dominant Hand)[E]	-0.222	-0.414	0.013	--	0.089	0.184	0.000	0.018	0.006	0.091	2.443

Movement Area (Non-Dominant Hand)^{E}	-0.072	0.001	0.012	--	0.154	0.135	0.000	0.021	0.013	0.157	2.410
**Movement Area Bias^{Q}**	**0.000**	**0.010**	**-0.006**	**--**	**-0.049**	**0.075**	**0.000**	**0.083**	**0.000**	**-0.040**	**2.538**
Note: superscripts E and Q mean an exponential or quadratic curve was fit to the date, respectively.											
